# Interaction of *Cutibacterium ( formerly Propionibacterium) acnes* with bone cells: a step toward understanding bone and joint infection development

**DOI:** 10.1038/srep42918

**Published:** 2017-02-20

**Authors:** Guillaume Ghislain Aubin, Marc Baud’huin, Jean-Philippe Lavigne, Régis Brion, François Gouin, Didier Lepelletier, Cédric Jacqueline, Dominique Heymann, Karim Asehnoune, Stéphane Corvec

**Affiliations:** 1 EA3826, Laboratory of Clinical and Experimental Therapeutics of Infections, IRS2, 22 Bd Benoni-Goullin, University of Nantes, Nantes, France; 2 Bacteriology and Hygiene Unit, CHU NANTES, France; 3 INSERM, UMR 957, Pathophysiology of Bone Resorption Laboratory and Therapy of Primary Bone Tumors, Medicine School, University of Nantes, Nantes, France; 4 CHU NANTES, Hôtel Dieu, Nantes, France; 5 INSERM, U1047, University of Montpellier, Nîmes, France; 6 Department of Microbiology, Caremeau University Hospital, Nîmes, France; 7 Clinique chirurgicale orthopédique et traumatique, CHU NANTES, Nantes, France; 8 Department of Oncology and Metabolism, Medical School, University of Sheffield, Sheffield, UK; 9 CRCINA, INSERM, Université d’Angers, Université de Nantes Nantes, France

## Abstract

*Cutibacterium acnes* (formerly *Propionibacterium acnes*) is recognized as a pathogen in foreign-body infections (arthroplasty or spinal instrumentation). To date, the direct impact of *C. acnes* on bone cells has never been explored. The clade of 11 *C. acnes* clinical isolates was determined by MLST. Human osteoblasts and osteoclasts were infected by live *C. acnes*. The whole genome sequence of six isolates of this collection was analyzed. CC36 *C. acnes* strains were significantly less internalized by osteoblasts and osteoclasts than CC18 and CC28 *C. acnes* strains (p ≤ 0.05). The CC18 *C. acnes* ATCC6919 isolate could survive intracellularly for at least 96 hours. *C. acnes* significantly decreased the resorption ability of osteoclasts with a major impact by the CC36 strain (p ≤ 0.05). Genome analysis revealed 27 genes possibly linked to these phenotypic behaviors. We showed a direct impact of *C. acnes* on bone cells, providing new explanations about the development of *C. acnes* foreign-body infections.

Bone is a mineralized tissue constantly remodeled under the simultaneous coordinated action of three cell types: bone matrix-resorbing osteoclasts, bone matrix-forming osteoblasts, and osteocytes embedded in the mineralized bone matrix^1^. This physiological process is tightly regulated and crucial to maintain a constant bone mass in adults. However, this balance can be impaired to favor resorption in pathological conditions, such as osteoporosis or bone and joint infection (BJI). The pathogenicity of *Cutibacterium acnes* has long been restricted to skin conditions^2^. However, *C. acnes* is increasingly recognized as a pathogen, mainly in foreign-body infections, such as arthroplasty or spinal instrumentation-associated infections^3^,^4^. The role of *C. acnes* in foreign-body infections is probably underestimated because, as an anaerobe, it needs an adapted bacterial transport system and usually a prolonged incubation time of up to 14 days for growth^5^. Late growth and/or growth only in enrichment media are often misinterpreted as contamination^6^. Recognition of the pathogenicity of *C. acnes* in such cases is dependent on its recovery in multiple, separately collected perioperative samples from a site expected to be sterile, in association with clinical symptoms^7^. Unfortunately, the clinical diagnosis of such low-grade infection can be challenging because these infections often mimic aseptic loosening with scarce or nonspecific clinical signs and symptoms^8^. The absence of a gold standard for the diagnosis and accurate criteria to define foreign-body infection may explain why *C. acnes* torpid infections are sometimes misclassified as aseptic loosening or mechanical failure^9^.

Therefore, the association between implant surgery and *C. acnes* infection is not always obvious^10^ and different studies have focused on population genetics and sequence typing in an attempt to identify sub-populations with different pathogenic potential^11^,^12^,^13^,^14^,^15^,^16^. To date, no clear association between phylotypes and infection/colonization has been reported. A genetic analysis of 210 *C. acnes* isolates from various origins demonstrated that phylotypes IB, II and III were more often associated with blood, cerebrospinal fluid and prosthetic hip infections whereas phylotype IA strains were highly associated with acne^13^. IB strains have also been more frequently isolated from infected prostheses than IA strains 17. Although several typing methods have been developed for *C. acnes*, whole genome-based typing reveals that only MLST methods have the discriminatory power to identify all distinct subtypes^13^. Recently, Yu *et al*. stated that studies focusing on well characterized types may further increase our understanding of the role of different *C. acnes* types in the pathogenesis of disease^18^.

Based on this statement, and because the direct impact of *C. acnes* isolates on bone cells has never been explored, we investigated the sequence type (ST) of *C. acnes* clinical isolates and developed different models in order to study the interaction between *C. acnes* and bone cells (osteoblasts or osteoclasts). Finally, whole genome sequencing (WGS) analysis revealed genetic tracks to explain the *C. acnes* behaviors observed.

## Material and Methods

### Bacterial strains

Eleven *C. acnes* clinical isolates (11 patients) were selected from a large collection of strains, including the main phylotypes and clonal complexes, mostly from previous studies^19^,^20^. Four isolates were recovered from monomicrobial spinal instrumentation infections, two from prosthetic monomicrobial infections, three from acne lesions and two were reference strains (ATCC11827 and ATCC6919). The characteristics of the selected isolates are summarized in Table 1. Implant-associated infections were confirmed using the Infectious Diseases Society of America criteria for bone and joint infections^21^.

### Molecular methods

#### Clonality

Multi-Locus Sequence Typing (MLST) was carried out on all isolates as described by Lomholt *et al*.^13^. This scheme is based on partial sequences of nine housekeeping genes comprising a total of 4,287 nucleotides and is available at http://pacnes.mlst.net/.

#### Whole genome sequencing

To investigate the relationship between phenotypic and genotypic results, WGS was performed on a subset from our collection. Five isolates were selected depending on their sequence type and clinical origin (Table 1). Bacterial DNA was extracted according to the manufacturer’s protocol using the commercial extraction kit QIAamp^®^ DNA mini kit (Qiagen, Hilden, Germany). Genome sequencing was performed using the MiSeq (Illumina) sequencing technology^22^. The genome alignment of *C. acnes* isolates was visualized using a progressive Mauve algorithm in the Mauve program version 20150226 build 10^23^. *C. acnes* genome sequences were annotated using the Rapid Annotation Subsystem Technology (RAST) automated web service^24^. Figure for genome comparison was generated by BRIG software^25^ and OAT software^26^.

### Co-culture with bone cells

Before infection of the bone cell cultures, strains were grown overnight at 37 °C in 40 mL of Brain Heart Infusion (BHI, Oxoid, Dardilly, France), harvested by centrifugation for 10 min at 800 *g* and washed once in 5 mL of phosphate-buffered saline (PBS). The pellets were suspended in 5 mL of Dulbecco’s Modified Eagle’s Medium (DMEM; Invitrogen-Life Technologies, Inc.). Bacterial suspensions were adjusted with a nephelometer to obtain 1 × 10^8^ CFU mL^−1^ and then diluted in DMEM.

#### Human osteoblast cultures

The human osteosarcoma cell line MG-63 purchased from ATCC was cultured in a 5% CO_2_ atmosphere at 37 °C in DMEM supplemented with 5% of fetal bovine serum (Hyclone Perbio, France). Three days before infection, cells were seeded in 24-well culture plates to obtain confluent monolayers. To confirm the results obtained with MG-63 cells, the same method was applied with two representative isolates (ATCC6919 and BL strains) on human mesenchymal stem cells (hMSC). hMSC were obtained from healthy donors. Blood draws were performed at the “Etablissement Français du Sang” (Nantes, France) after obtaining the informed consent of all healthy donors. The experimental protocol was approved by the ethics committee of the Medical School of Nantes. All experiments were performed in accordance with the Good Scientific Practice guidelines of the Medical School of Nantes and all relevant guidelines and regulations. hMSC were cultured in DMEM supplemented with 10% of fetal bovine serum, 1 ng/mL of basic Fibroblast Growth Factor (bFGF; R&D systems, UK), 100 U/mL of penicillin/streptomycin, and 2 mM L-glutamine. Adherent cells were frozen at passage 2 after characterization by flow cytometry (CD45−, CD34−, CD105+, CD73+, and CD90+, purity ≥99%) prior to further experiments^27^.

#### Internalization assay

Bacterial internalization experiments were adapted from Crémet *et al*. study^28^. Briefly, the MG-63 monolayers were infected for 2 h (multiplicity of infection (MOI) of 100:1). The number of bacteria present in the inocula was checked by plating dilutions on Schaedler plates incubated under anaerobic conditions. After 2 h of infection, cells were washed twice with PBS and incubated for a further 2 h in 1 mL of DMEM containing 1% of penicillin/streptomycin to kill the remaining extracellular bacteria. The cell monolayers were then washed three times and lysed with 0.1% Triton X-100. The lysates were serially diluted and plated on Schaedler plates to count the number of intracellular bacteria. The results were expressed as percentages of inoculum.

#### Bacterial internalization assessment by cell staining and confocal microscopy

To confirm the bacterial internalization, MG-63 cells were seeded onto slides after the internalization assay and then Gram and Giemsa stained. For live confocal microscopy, MG-63 cells were seeded onto Ibidi^®^ μ-Slide 18 well (Munich, Germany) and infected with two selected clinical strains (ATCC6919 and BL), as described above. Fluorescein isothiocyanate (Sigma-Aldrich^®^, Saint Quentin Fallavier, France) was used to visualize the bacteria, along with calcein red/orange (Thermo Fischer Scientific, Waltham, MA USA) to label the cellular membrane. Slides were acquired on a Nikon A1 Rsi confocal microscope designed for live cell imaging using an Okolab environmental chamber to regulate temperature and air/CO_2_/N_2_. Pictures were analyzed with Fiji software^29^. 3D images were processed with NIS elements (Nikon Instruments Inc.) and Volocity 3D Image Analysis Software (PerkinElmer).

#### Persistence assay

ATCC6919 and BL *C. acnes* strains were internalized in MG-63 cells as described above. Following incubation with penicillin/streptomycin, MG-63 cells were lysed at 12, 24, 48, 72, 96 and 168 h post-infection to assess intracellular bacterial viability and persistence. Lysates were seeded on Schaedler plates. Bacterial growth was observed after five days of incubation at 37 °C under anaerobic atmosphere conditions.

#### Cell viability and osteoclastogenesis assay

The generation of osteoclasts from human CD14^+^ monocytes has been described previously^30^. Briefly, purified CD14^+^ cells from different healthy donors were cultured in α-MEM with 10% FCS and 25 ng/mL human M-CSF (R&D systems). Blood draws were performed at the “Etablissement Français du Sang” (Nantes, France) after obtaining the informed consent of all healthy donors. The experimental protocol was approved by the ethics committee of the Medical School of Nantes. All experiments were performed in accordance with the Good Scientific Practice guidelines of the Medical School of Nantes and all relevant guidelines and regulations. At day 3, cells were infected with *C. acnes* as described above (MOI 10:1), followed by antibiotic treatment for 2 h and addition of 100 ng/mL RANKL. Multinucleated cells formed with 3 or more nuclei were counted after TRAP staining (Sigma, France). Cell growth was measured using the crystal violet assay as described previously^30^.

#### Bone resorption assay

Mature osteoclasts were obtained by differentiation of CD14^+^ cells and then infected by *C. acnes* as described above (MOI 10:1), followed by antibiotic treatment for 2 h. Next, cells were incubated for 48 h on 96-well Corning Osteo Assay^®^ plates (OsteoCorning, Corning, MA). After the culture period, cells were removed with bleach solution and the area of resorption pits was quantified using Image J software (NIH, Bethesda, MD).

## Results

### *C. acnes* belonging to clonal complex 36 are less internalized by human osteoblast-like cells

**Figure 1:**
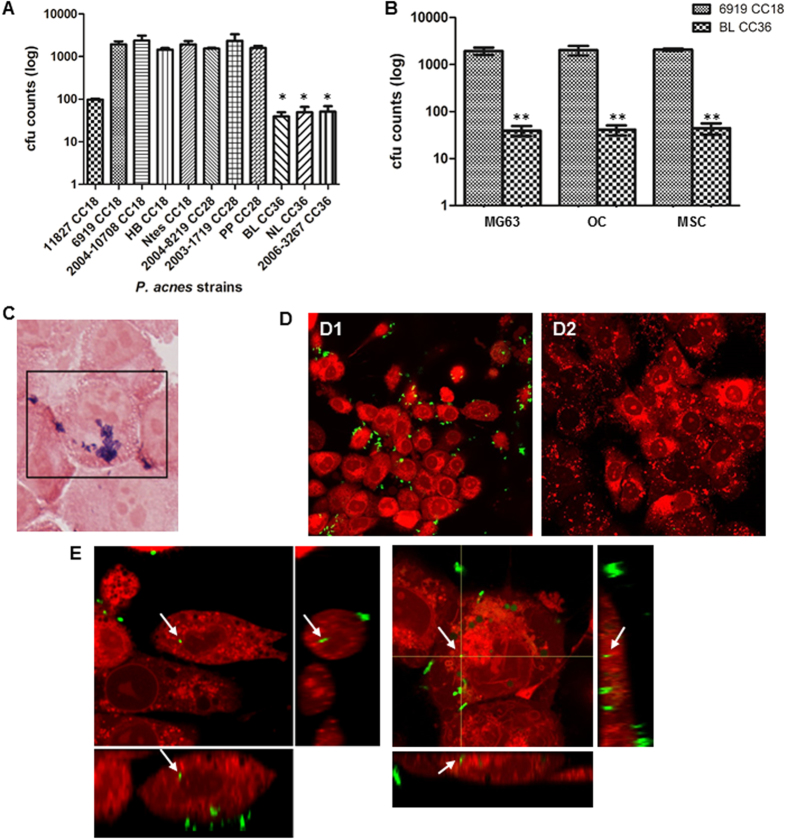
Host cell type-specific internalization of *Cutibacterium acnes.* (**A**) MG-63 cells were infected with different *C. acnes* strains at a MOI of 100:1 for 2 hours. After 2 h, cells were washed, treated with penicillin/streptomycin, washed again and lysed. CFU counts were determined after 5 days of anaerobic culture of the lysate on Schaedler plates. *P < 0.01. (**B**) MG-63 cells, osteoclasts (OC) and mesenchymal stem cells (MSC) were infected with different *C. acnes* strain at a MOI of 100:1 for 2 hours. After 2 h, cells were washed, treated with penicillin/streptomycin, washed again and lysed. CFU counts were determined after 5 days of anaerobic culture of the lysate on Schaedler plates. **P < 0.01. (**C**) MG-63 cells Gram stained (ATCC6919 *C. acnes* cells appear in purple). (**D**) Live confocal microscopy pictures after 2 h of incubation with MG-63 cells and washing. MG-63 cell membranes were stained with calcein red/orange (red). *C. acnes* bacteria were stained with fluorescein isothiocyanate (green). D1. Result with *C. acnes* ATCC6919 strain. D2. Result with *C. acnes* BL isolate. (**E**) Orthogonal views from different planes (x/y, x/z or y/z) of the live confocal microscopy images used to analyze the ATCC6919 CC18 *C. acnes* location. Bacteria (FITC, green) inside the cell (cellular membranes, stained with calcein red/orange, appear in red) are indicated by a white arrow.

The bacterial internalization assay by osteoblasts was carried out using 11 *C. acnes* strains. Clonal complexes of these isolates are shown in Table 1. CC36 *C. acnes* strains were less invasive than CC18 and CC28 *C. acnes* strains toward osteoblasts (mean percentage of internalized bacteria <0.01% for CC36 *P. acnes* strains *versus* more than 1% for CC18 and CC28 *C. acnes* strains (Fig. 1A)). The ATCC11827 CC18 *C. acnes* strain exhibited a similar invasiveness to CC36 isolates (Fig. 1A). To assess whether the observed results were not related to the nature of osteosarcoma MG-63 cells, we confirmed our results using mesenchymal stem cells. These had the advantage of sharing common features with all bone cells and enabled the results observed to be extrapolated to all derivative cells. The mean percentages obtained previously were similar for both isolates: CC18 ATCC6919 and CC36 BL *C. acnes* (Fig. 1B). Using these two isolates, the same results were also observed for the interaction, especially with osteoclasts (Fig. 1B). Determination of bacterial subcellular localization by Gram staining (Fig. 1C) and confocal microscopy (after stringent washing to remove unattached bacteria) confirmed the intracellular presence of *C. acnes* and the discrepancies observed between the clonal complexes (Fig. 1D,E, Supplementary data video 1).

### *C. acnes* can survive in human osteoblast cells

**Figure 2:**
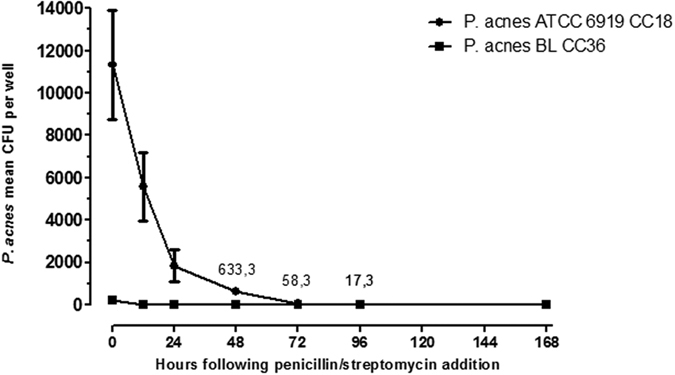
Persistence and viability of *C. acnes* strain ATCC6919 (CC18) and *C. acnes* BL (CC36) after internalization by MG-63 osteosarcoma cells. The mean CFU for three independent experiments for each time point is shown. Vertical lines represent the 95% confidence interval.

Low-grade and chronic infections can be due to bacteria hidden from the immune response, inside cells. Herein, as *C. acnes* isolates demonstrated different osteoblast internalization abilities, we further evaluated whether CC18 ATCC6919 and CC36 BL *C. acnes* strains underwent clearance by osteoblasts. As shown in Fig. 2, the CC18 ATCC6919 strain could survive in osteoblasts after 96 h of incubation. A significant decrease in the number of viable intracellular bacteria was observed during the course of the experiment (p = 0.0007). The mean CFU/well ranged from 1.15 × 10^4^ at time 0 to 17.3 after 96 h of incubation. No colony remained at Day 7. In contrast, no colony remained at 24 h with the CC36 BL strain. These results demonstrate the ability of *C. acnes* strains to remain viable intracellularly at least 96 h after infection, in our experimental model.

### *C. acnes* infection has no impact on osteoclastogenesis

**Figure 3:**
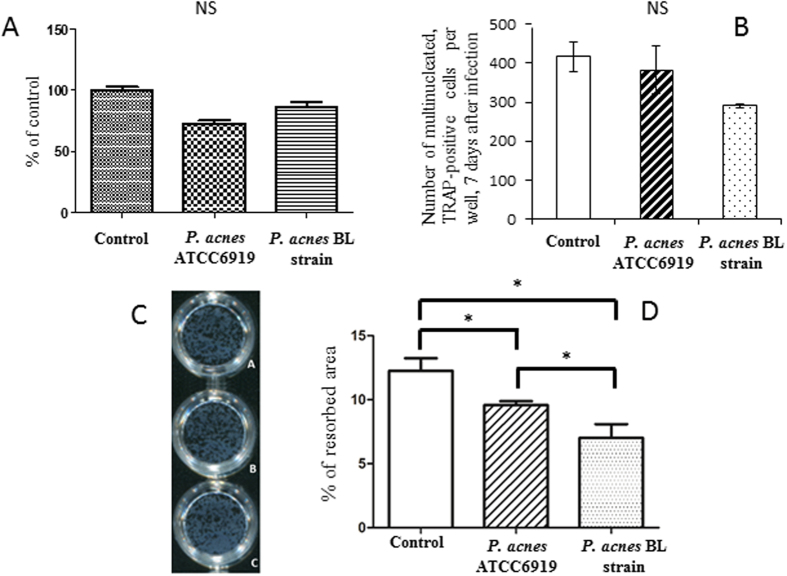
Impact of *Cutibacterium acnes* on the osteoclast lineage. (**A**) Cell viability after infection of human CD14+ monocytes by *C. acnes*. NS: not significant. (**B**) Effect of infection of human CD14+ monocytes by *C. acnes* on osteoclastogenesis. Multinucleated cells formed with 3 or more nuclei (osteoclasts) were counted after TRAP staining. NS: not significant. (**C**) Resorption pit area due to mature osteoclasts uninfected (Ca), infected by *C. acnes* ATCC6919 (Cb), and infected by *C. acnes* BL (Cc). (**D**) Percentage of matrix resorbed by mature osteoclasts uninfected (control), or infected by *C. acnes* after 48 hours of culture. *P < 0.05 (one-tailed t-test).

We then evaluated whether bacterial infection of the precursors could impact osteoclastogenesis. Cell viability was not significantly changed in any of the subgroups (Fig. 3A). Human CD14+ monocytes were cultured in control conditions or in the presence of ATCC6919 or CC36 BL *C. acnes* for 2 h. Seven days after internalization of ATCC6919 or CC36 BL *C. acnes*, a mean (±SD) of 382 ± 100 and 290 ± 6 of the cells, respectively, were multinucleated and TRAP-positive, compared with uninfected cells (416 ± 67) (Fig. 3B).

### *C. acnes* strains inhibit osteoclast bone resorption capacities

We then decided to evaluate the impact of *C. acnes* infection on mature osteoclast function. The mean percentage of the resorbed area (±SD) by cells infected with CC18 ATCC6919 or CC36 BL *C. acnes* strains was 9.6 ± 0.53% and 7 ± 1.9%, respectively. A significant difference (p < 0.05) was observed between uninfected wells (12 ± 1.7%) and *C. acnes* infected wells after 48 h of resorption. Moreover, CC36 *C. acnes* showed a more significant ability to decrease the resorption ability of osteoclasts than the CC18 ATCC6919 strain (p < 0.05). The results of the most representative experiment are presented in Fig. 3C,D.

### Whole genome sequencing

**Figure 4:**
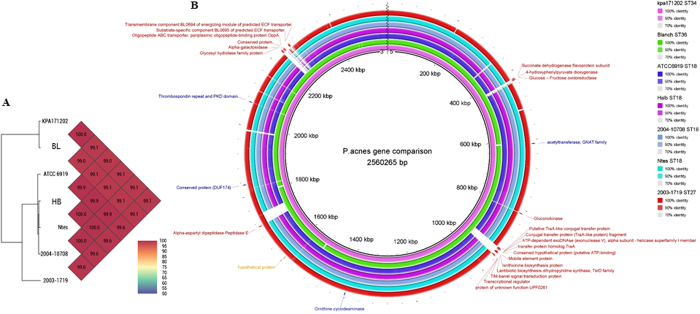
Core and pan-genome analysis of *Cutibacterium acnes* strains. (**A**) General comparison of the whole genome sequence of *C. acnes* generated with OrthoANI values calculated from OAT (Orthologous Average Nucleotide Identity Tool) software (http://www.ezbiocloud.net/app). (**B**) Comparison of *C. acnes* genomes. The RAST-annotated genomes of 6 *C. acnes* isolates (ATCC6919, HB, 2003-1719, 2004-10708, BL and Ntes) were aligned using *C. acnes* KPA171202 as a reference sequence. The genomes were aligned and the figure was created with BRIG software (http://brig.sourceforge.net/). Red arrows outside the outer circle indicate the locations of non-internalized strain-specific genes, blue arrows indicate the locations of internalized strain-specific genes.

WGS was performed on a subset of isolates to highlight candidate genes potentially involved in the differences in phenotypic behavior observed previously (Table 1). The ATCC6919, BL, Ntes, HB, 2003-1719 and 2004-10708 strains yielded 2617, 2469, 2416, 2410, 2463 and 2410 predicted coding sequences (CDSs), respectively^31^,^32^. Slightly less than half (44 to 47%) of the CDSs in each strain could be functionally categorized into 315 to 331 subsystems. Among the subsystems, carbohydrates, “cofactors, vitamins, prosthetic groups, pigments”, “amino acids and derivatives” and protein metabolism had the greatest number of genes. An overview of the genome characteristics of the isolates and their subsystem statistics is shown in Table 1 of the supplementary data. The SEED viewer identified 27 genes that were specific to the non-internalized isolates and 6 genes specific to the internalized isolates (Fig. 4, Table 2 in the supplementary data).

## Discussion

Most studies on the interaction between bacteria and bone cells during foreign-body infection have focused on the *Staphylococcus aureus* model, the main pathogen isolated in this context^33^,^34^,^35^. Very few other species have been studied and little is known about the impact of *C. acnes* either in aseptic loosening or low-grade infection^36^. In fact, *C. acnes* host-pathogen interactions have only been well demonstrated in the acne field, with a specific cutaneous innate immunity pattern according to phylotype^37^. To the best of our knowledge, this study is the first to provide information about the interaction and relationship between *C. acnes* and bone cells, in line with clinical and molecular typing data. Herein, using different lineages of *C. acnes*, we showed that strains belonging to the lineage previously associated with PJI (phylotype IB, CC36) were significantly less internalized by bone cells than those belonging to the lineage previously associated with acne or spine instrumentation infections (phylotype IA, CC18/CC28)^13^,^17^. We also showed that *C. acnes* can decrease the resorption ability of osteoclast with a greater effect of *C. acnes* belonging to CC36.

**Figure 5:**
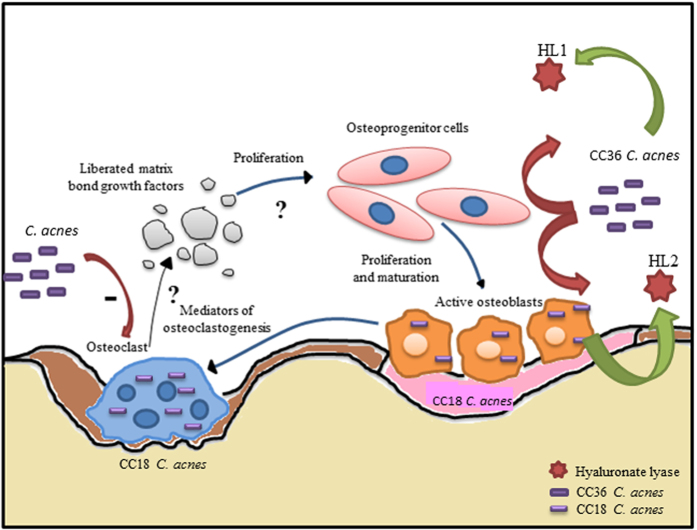
Schematic representation of the impact of *Cutibacterium acnes* on bone cells. Depending on the clonal complexes, there are two ways for *C. acnes* to interact with bone cells. On one hand, CC18 *C. acnes* can be internalized by osteoblasts and osteoclasts. On the other hand, CC36 *C. acnes* are less internalized. Moreover, infection of mature osteoclasts by *C. acnes* decreases their bone resorption ability with a major impact by CC36 isolates. A question mark highlights the remaining issues to understand better the interaction between *C. acnes* and bone cells.

As shown in Fig. 5, both osteoblast and osteoclast interactions were studied to take into account the overall impact of this bacterium on the bone remodeling process. Osteoblasts, derived from mesenchymal bone marrow precursors, control this process by synthesizing the components of the bone matrix and by modulating the activity of the bone-resorbing osteoclasts^1^. Although bone cells can internalize bacteria by different mechanisms, *i.e.*, phagocytosis for osteoclasts or endocytosis for osteoblasts^38^,^39^, interestingly, we observed a similar internalization behavior in response to *C. acnes* infection with osteoblasts, human mesenchymal stem cells (precursors of osteoblasts derived from bone marrow) and osteoclasts.

Using different epithelial cells, Mak *et al*. showed that a ST33/CC36 *C. acnes* strain (isolated from a prostatectomy tissue sample) was more internalized in RWPE1 cells than in HaCaT and HEKa cells^40^. Unfortunately, this could not be compared with our internalization level because only a relative invasion percentage was provided. On the other hand, two CC36 strains (P6 from cancerous prostate and KPA171202) and one CC18 strain (P266, pleuropulmonary isolate) showed strain-dependent differences in their uptake by the THP-1 cell line (macrophage model)^41^, without any correlation to the clade (45.9% and 8.9% of the initial inoculum for KPA and P6 strains, respectively). Taken together, these studies suggest that the CC36 strain trait observed is linked to the bone origin of the cells used in our study.

From a pathophysiological point of view, the evasion mechanism observed for CC36 *C. acnes* isolates could allow this clade to leave the site of infection, disseminate into deeper tissue layers and cause PJI (*i.e.*, arthroplasty infection). Inside the deeper tissue, close to the material, the local immune defect fosters the low-grade infections observed^42^. In contrast, for CC18 and CC28 clades, mostly involved in spinal instrumentation infection, the internalization process observed could allow them to escape from the numerous immune cells located under the skin^43^ and generate an infection locally, favored by the spine instrumentation close to the skin. Moreover, the failure of osteoblasts to eradicate the intracellular CC18 *C. acnes* clade, resulting in bacterial persistence in the host, could explain the clinically observed BJI chronicity.

Additionally, we showed that direct infection of mature osteoclasts with *C. acnes* decreases their bone resorption ability. *In vivo*, a decrease in bone resorption will unbalance the bone remodeling process, leading to the possible aseptic loosening observed during *C. acnes* infection^44^. Interestingly, the CC36 *C. acnes* clade showed a major impact on osteoclast bone resorption ability. This result constitutes another argument to explain the deeper infection (PJI) tropism of CC36 *C. acnes* clades.

Finally, WGS analysis confirmed the high genome coverage and the highly stable chromosome previously observed^45^,^46^. All indels observed and linked to clade features (and most likely internalization) were recently described by Scholz *et al*.^45^. We identified 27 genes possibly linked to the behaviors observed (Table 2 in supplementary data). Perhaps the best candidate to explain our results is the *hyaluronate lyase* gene (indel_14)^45^. Indeed, the study of Tyner *et al*.^47^ and the recent communication from Nunes *et al*.^48^ highlighted that the allele diversity in the hyaluronate lyase gene could lead to different enzyme activities modulating, *de facto*, the penetration of *C. acnes* into tissues rich in hyaluronic acid (*e.g.* joints).

In conclusion, our study provides new data about *C. acnes* interactions with bone cells. Its strength is that it addresses the two main agents of the bone remodeling process with bacteria perfectly genetically defined due to the most recent molecular tools. We clearly identify two *C. acnes* behaviors depending on their genetic background (CC). These two behaviors provide explanations about the development of *C. acnes* PJI or spine instrumentation infection.

## Additional Information

**How to cite this article:** Aubin, G. G. *et al*. Interaction of *Cutibacterium (formerly Propionibacterium) acnes* with bone cells: a step toward understanding bone and joint infection development. *Sci. Rep.* **7**, 42918; doi: 10.1038/srep42918 (2017).

**Publisher's note:** Springer Nature remains neutral with regard to jurisdictional claims in published maps and institutional affiliations.

## Supplementary Material

Supplementary Data

Supplementary Video 1

## Figures and Tables

**Table 1:** Characteristics of *Cutibacterium acnes* selected for whole genome sequencing and internalization assay.

Isolate name	Clinical source	Clonal complex MLST	Phylotype (*recA* **type)**	Internalization level
ATCC11827	Acne	CC18	IA	<0.01%
ATCC6919*^¥^	Acne	CC18	IA	>1%
PA PP	Acne	CC28	IB	>1%
PA HB*	Acne	CC18	IA	>1%
PA NL	Acne	CC36	IB	<0.01%
PA 2004-8219	Spine	CC28	IB	>1%
PA 2006-3267	Spine	CC36	IB	<0.01%
PA 2003-1719*	Spine	CC28	IB	>1%
PA 2004-10708*	Spine	CC18	IA	>1%
PA BL*^¥^	Hip prosthesis	CC36	IB	<0.01%
PA Ntes*	Knee prosthesis	CC18	IA	>1%

^*^Isolates selected for WGS.

^¥^Isolates selected for mesenchymal stem cell internalization assay.
