# Supplementary material for: Interaction of Cutibacterium ( formerly Propionibacterium) acnes with bone cells: a step toward understanding bone and joint infection development
Source: Sci Rep. 2017 Feb 20;7:42918. doi: 10.1038/srep42918 (PMC5317161; doi:10.1038/srep42918)
Supplement: Supplementary Data [file srep42918-s1.pdf]

Title page

Interaction of *Cutibacterium (formerly Propionibacterium) acnes* with bone cells: a step toward understanding bone and joint infection development

Guillaume Ghislain Aubin[1,2]

[1] EA3826, Laboratory of Clinical and Experimental Therapeutics of Infections, IRS2, 22 Bd Benoni-Goullin, University of Nantes, Nantes, France.

[2] Bacteriology and Hygiene Unit, Nantes University Hospital, France.

Marc Baud'huin[3,4]

[3] INSERM, UMR 957, Pathophysiology of Bone Resorption Laboratory and Therapy of Primary Bone Tumors, Medicine School, University of Nantes, Nantes, France.

[4] Nantes University Hospital, Hôtel Dieu, Nantes, France.

Jean-Philippe Lavigne[5,6]

[5] INSERM, U1047, University of Montpellier, Nîmes, France.

[6] Department of Microbiology, Caremeau University Hospital, Nîmes, France.

Régis Brion[3,4]

[3] INSERM, UMR 957, Pathophysiology of Bone Resorption Laboratory and Therapy of Primary Bone Tumors, Medicine School, University of Nantes, Nantes, France.

[4] Nantes University Hospital, Hôtel Dieu, Nantes, France.

François Gouin[3,7]

[3] INSERM, UMR 957, Pathophysiology of Bone Resorption Laboratory and Therapy of Primary Bone Tumors, Medicine School, University of Nantes, Nantes, France.

[7] Clinique chirurgicale orthopédique et traumatique, Nantes University Hospital, Nantes, France.

Didier Lepelletier[1,2]

[1] MiHAR Lab, IRS2, 22 Bd Benoni-Goullin, University of Nantes, Nantes, France.

[2] Bacteriology and Hygiene Unit, Nantes University Hospital, France.

Cédric Jacqueline[1]

[1] EA3826, Laboratory of Clinical and Experimental Therapeutics of Infections, IRS2, 22 Bd Benoni-Goullin, University of Nantes, Nantes, France.

Dominique Heymann[3,4,8]

[3] INSERM, UMR 957, Pathophysiology of Bone Resorption Laboratory and Therapy of Primary Bone Tumors, Medicine School, University of Nantes, Nantes, France.

[4] Nantes University Hospital, Hôtel Dieu, Nantes, France.

[8] Department of Oncology and Metabolism, Medical School, University of Sheffield, Sheffield, UK.

Karim Asehnoune[1]

[1] EA3826, Laboratory of Clinical and Experimental Therapeutics of Infections, IRS2, 22 Bd Benoni-Goullin, University of Nantes, Nantes, France.

Stéphane Corvec[1,2,9*]

[1] EA3826, Laboratory of Clinical and Experimental Therapeutics of Infections, IRS2, 22 Bd Benoni-Goullin, University of Nantes, Nantes, France.

[2] Bacteriology and Hygiene Unit, Nantes University Hospital, France.

[9] INSERM U892 - CNRS 6299 CRCNA Centre Régional en Cancérologie Nantes Angers - University of Nantes, Nantes, France. Team 2: Clinical and translational research in skin cancer.

Table 1 Supplementary data. *Cutibacterium acnes* genomes overview

	Reference strain	Strains					
	KPA171202 (DSM 16379)	ATCC6919 (DSM 1897)	BL	Ntes	HB	2003-1719	2004-10708
Sequence type	ST34	ST18	ST36	ST18	ST18	ST27	ST18
Clinical source	contamination of an anaerobic culture	Acne	Hip arthroplasty	Knee arthroplasty	Acne	Spine	Spine
Size (Mb)	2.6	2.6	2.5	2.5	2.5	2.5	2.5
%GC	60	60.2	60	60	60	60.1	60.1
Number of ORFs							
Number of RNAs	63	53	45	48	48	49	48
Subsystem coverage (%)	44	47	47	47	47	46	47
Number of subsystems	315	330	331	322	324	327	324
**Subsystem feature counts**							
Cofactors, vitamins, prosthetic groups, pigments	255	202	189	183	185	180	183
Cell wall and capsule	45	74	74	73	73	74	73
Virulence, disease and defense	31	33	31	30	30	31	31
Potassium metabolism	19	14	13	13	3	13	13
Photosynthesis	0	0	0	0	0	0	0
Miscellaneous	32	22	20	20	20	20	20
Phages, prophages, transposable elements, plasmids	0	18	5	0	0	6	0
Membrane transport	33	46	46	41	41	42	41
Iron acquisition and metabolism	4	4	5	4	4	4	4
RNA metabolism	105	93	85	85	89	85	85
Nucleosides and nucleotides	88	103	91	94	93	91	94
Protein metabolism	182	227	215	216	215	215	216
Cell division and cell cycle	19	23	23	23	24	23	23
Motility and chemotaxis	0	3	3	3	3	3	3
Regulation and cell signaling	32	17	17	17	17	17	17
Secondary metabolism	2	0	2	0	0	0	0
DNA metabolism	50	68	69	64	63	64	63
Fatty acids, lipids, and isoprenoids	59	101	77	87	86	86	86
Nitrogen metabolism	17	22	16	23	19	19	21
Dormancy and sporulation	2	2	2	2	2	2	2
Respiration	91	97	98	96	95	94	94
Stress response	38	49	50	48	47	48	51
Metabolism of aromatic compounds	1	1	1	1	1	1	1
Amino acids and derivatives	210	288	261	267	266	272	266
Sulfur metabolism	11	13	14	11	11	11	11
Phosphorus metabolism	28	37	29	29	29	29	29
Carbohydrates	227	360	323	331	332	321	333

Table 2 supplementary data. *Cutibacterium acnes* gene comparison according to phenotypic features observed (bone cells internalization).

	Non-internalized strains specific genes	Internalized strains specific genes
Total	22	5
Not part of a subsystem	11	1
	Succinate dehydrogenase flavoprotein subunit	acetyltransferase, GNAT family
	Glucose – Fructose oxidoreductase	Ornithine cyclodeaminase
	4-hydroxyphenylpyruvate dioxygenase	Streptomyces scabies esterase domain
	Gluconokinase	Conserved protein (DUF174)
	Putative TraA-like conjugal transfer protein	Thrombospondin repeat and PKD domain
	conjugal transfer protein (TraA-like protein) fragment	
	ATP-dependent exoDNAse (exonuclease V), alpha subunit - helicase superfamily I member	
	transfer protein homolog TraA	
	conserved hypothetical protein (putative ATP-binding)	
	Mobile element protein	
	Lanthionine biosynthesis protein LanB	
	Lantibiotic biosynthesis dihydropyridine synthase, TsrD family	
	TIM-barrel signal transduction protein	
	Transcriptional regulator	
	protein of unknown function UPF0261	
	Alpha-aspartyl dipeptidase Peptidase E	
	Glycosyl hydrolase family protein	
	Alpha-galactosidase	
	Conserved protein	
	Oligopeptide ABC transporter, periplasmic oligopeptide-binding	

	protein OppA	
	Substrate-specific component BL0695 of predicted ECF transporter	
	Transmembrane component BL0694 of energizing module of predicted ECF transporter	

*PSI-Blast iteration: No putative conserved domains have been detected

Video legend

Cutibacterium acnes ATCC6919 inside MG-63 osteoblast cell
